# Field Application of a Subunit Vaccine against an Enteric Protozoan Disease

**DOI:** 10.1371/journal.pone.0003948

**Published:** 2008-12-16

**Authors:** Michael G. Wallach, Udi Ashash, Amnon Michael, Nicholas C. Smith

**Affiliations:** 1 Institute for the Biotechnology of Infectious Diseases, University of Technology Sydney, Broadway, Australia; 2 ABIC Biological Laboratories Teva Ltd., Beit Shemesh, Israel; Columbia University, United States of America

## Abstract

**Background:**

Coccidiosis is a major global veterinary health problem in intensively reared chickens. It is caused by apicomplexan parasites of the genus *Eimeria*.

**Principal Findings:**

A subunit vaccine composed of purified antigens from the gametocytes of *Eimeria maxima* was used to stimulate the production and transfer of maternal antibodies between breeding hens and their hatchlings. The vaccine was injected into hens twice before they began laying eggs. Immunization had no adverse affects on egg laying or health of the hens and resulted in high antibody levels throughout the life of the hens. Progeny of immunized hens excreted significantly less oocysts of various species of *Eimeria* in their faeces than chicks from unvaccinated hens. Furthermore, the offspring of vaccinated hens developed stronger natural immunity to *Eimeria*, so that they were resistant to challenge infection even at 8 weeks of age, well after all maternal antibodies had left their circulation. Field trials were conducted in South Africa, Brazil and Thailand, involving at least 1 million progeny of vaccinated hens and at least 1 million positive control birds (raised on feed containing anticoccidial drugs or immunized with a live vaccine) in each country. Additionally, trials were carried out in Israel involving 60 million progeny of vaccinated hens and 112 million positive control birds. There were no significant differences in growth rate, feed conversion ratios or mortality in the offspring of vaccinated hens compared with the positive control chickens in any of these countries regardless of different management practices, different breeds of chickens or climate.

**Conclusions:**

These results demonstrate that a vaccine composed of antigens purified from the gametocytes of *Eimeria* can be used safely and effectively to prevent the deleterious effects of coccidiosis. It is the first subunit vaccine against any protozoan parasite to be successfully applied on a commercial scale.

## Introduction

Coccidiosis is a major global veterinary health problem impacting on the production of domesticated sheep, cattle, poultry and even fish. It is a particularly problematic disease in chickens (due to their intense rearing conditions), costing the industry £38 million per year in the UK alone and, extrapolating from this, over 2 billion US dollars to the world's farmers and poultry industries annually [1]. It is caused by various species of the genus, *Eimeria*, which are ubiquitous parasites of the Phylum Apicomplexa. In chickens, there are several species that cause the disease, the three most important being *Eimeria tenella*, *Eimeria maxima* and *Eimeria acervulina*. Infection with *Eimeria* begins with the ingestion of sporulated oocysts, which are found in the floor litter of any typical poultry house. Sporozoites are liberated from the oocysts and rapidly invade the host intestinal epithelium, commencing the first of several asexual rounds of reproduction that rapidly amplify the number of parasites infecting individual birds. Ultimately, the asexual parasites (merozoites) develop into macro- and microgametocytes, the latter fertilising the former to produce oocysts, which are shed in the faeces of chickens, contaminating the environment of whole flocks of birds. For each oocyst ingested by a naive chicken, several hundred thousand new oocysts are produced. The oocyst possesses an extremely hardy protective wall – the oocyst wall – that protects the parasites contained within it and facilitates their successful transmission from one host to the next [2]. The oocyst wall originates from the fusion of specialized organelles – the wall forming bodies – found in the macrogametes of the parasite [3]. We have previously provided evidence that proteins within these organelles are processed and cross-linked via dityrosine bonds to form the essential structure of the oocyst wall [4]. Two of the key proteins of this process are gam56 and gam82, the main components of a vaccine comprised of antigens from the gametocytes of *Eimeria maxima* [4]–[6]. It is believed that antibodies stimulated by vaccination interfere with the formation of cross-links between these proteins and, hence, inhibit oocyst wall formation, effectively interrupting the parasite's lifecycle at the transmission stage [2], [4]–[6].

We [4]–[7] and others [8]–[10] have previously shown that immunization of breeding hens – either with purified gametocyte antigens or via deliberate infection with *E. maxima* – results in the passive maternal transfer of large quantities of anti-parasite IgG from hen to egg yolk and, hence, to young chicks, protecting those chicks against infection. In laboratory-controlled conditions, the level of protection is very high, with complete abrogation of oocyst shedding being observable [5]–[7]. This maternal immunity is able to give protection against multiple species of *Eimera* [11], unlike direct immunity, which is species-specific [12], [13]. Furthermore, in floor pen trials designed to mimic field conditions, chicks originating from vaccinated hens shed 60–70% fewer oocysts over their lifetime than did chicks from unvaccinated hens [14]. This resistance to infection outlasts the life of maternal antibodies in the growing birds and is presumed to be a result of the fact that maternal immunity induced by vaccination reduces, without totally eliminating, transmission of oocysts between birds, allowing individual birds to develop their own natural, anti-asexual stage immunity on top of the maternal immunity arising from vaccination with gametocyte antigens. (Anti-asexual stage immunity to *Eimeria* has long been recognised to be extremely strong and effective and is the basis for the success of attenuated live vaccines against coccidiosis [12], [13], [15]). This explanation for the effectiveness of maternal immunization with purified gametocyte antigens has never been tested formally. Furthermore, to date, the commercial performance (ie weight gain, feed conversion, survival) of the progeny of vaccinated hens has not been reported. Thus, in the experiments reported here, we examined the effect of vaccination with purified gametocyte proteins on (i) the health and egg production of breeding hens, (ii) antibody levels in commercial breeding hens, (iii) reduction of parasite reproduction in the offspring of vaccinated hens at a variety of times after hatching, and (iv) weight loss, feed conversion rate and mortality caused by challenge infection with several species of *Eimeria*.

## Results and Discussion

### Safety and immunogenicity of vaccination with purified gametocyte antigens in breeding hens

We evaluated the performance of breeder hens (Cobb, Ross, ANAK and others) vaccinated with purified gametocyte antigens in broiler breeder farms around the world. Breeding hens were vaccinated twice prior to the start of their laying period (ie, at 15 and 20 weeks of age). We tested the null hypotheses that immunization with purified gametocyte antigens has no deleterious effects on hen mortality or egg production. The hens suffered no adverse reactions, no damage at the site of injection, and no impairment of performance as a result of vaccination with purified gametocyte antigens. Furthermore, in every farm where these trials were conducted, the survival rate and the number of eggs laid was not significantly different for vaccinated versus unvaccinated, control hens (Table 1). Thus, we must accept the null hypothesis that immunization does not have any adverse affects on maternal health or egg production. Indeed, on several farms there actually appeared to be a slight (albeit not statistically significant), unanticipated improvement in the performance of breeding flocks that were vaccinated. Furthermore, the total number of chicks hatched per hen was improved. In, for example, the Argentine trial 119 eggs hatched in the vaccinated group vs. 116 in the control group, for a total of 13,473 extra live chicks from the 4,646 vaccinated hens versus the 4,669 unvaccinated hens.

**Table 1:** The effect of vaccination with purified gametocyte antigens of *Eimeria maxima* on mortality and egg production by breeding hens.

Country	Number of hens	Mortality (%)	Eggs laid per hen
**Argentina (Cobb Breed)**			
Purified gametocyte antigen	4,646	9.08%	152
Control	4,669	17.41%	149
**Brasil (Cobb Breed)**			
Purified gametocyte antigen Farm 1	19,854	15.58%	177
Control Farm 1	19,455	17.82%	177
Purified gametocyte antigen Farm 2	20,100	11.21%	176
Control Farm 2	20,218	14.96%	170
Purified gametocyte antigen Farm 3	18,747	4.09%	192
Control Farm 3	18,734	4.14%	188
Purified gametocyte antigen Farm 4	20,284	9.93%	180
Control 4	19,194	13.14%	178
**India (Ross Breed)**			
Purified gametocyte antigen	3,500	7.9%	182
Control	3,500	10.4%	175

Breeding hens were injected intramuscularly with 50 µg of purified gametocyte antigens emulsified in 0.5 ml of water-in-oil adjuvant at 15 and 20 weeks of age. Mortality rates and egg production of the flocks of hens were monitored until the end of their productive lives (around 60–65 weeks of age). Neither mortality or egg production were affected by vaccination (one-way ANOVA).

**Figure 1:**
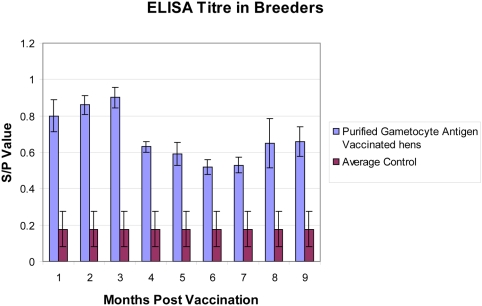
Effect of immunization with purified gametocyte antigens of *Eimeria maxima* on gametocyte antigen-specific antibody levels in flocks of breeding hens. Commercial broiler breeder hens were vaccinated twice with purified gametocyte antigens (PGA) at 15 and 20 weeks of age. Sera (10–15 samples per flock) were collected at each time point post vaccination from 10 flocks of chickens (six from Israel, two from South Africa, and one each from Argentina and Thailand). The ELISA results are expressed as an S/P ratio, which is calculated as follows: (Sample optical density value – Negative control optical density value)/(Positive control optical density value – Negative control optical density value). Results show the average S/P±Standard Error for the ten flocks at different times post-vaccination. The average S/P for four control flocks from some of the same farms that were tested during the testing period are also shown.

Hens were bled and ELISA carried out at various time points post-vaccination. The commercial ELISA kit contains 96-well plates coated with affinity purified gametocyte antigens, as well as positive and negative control sera. The ELISA test results are recorded as an S/P ratio, calculated as follows: (Sample optical density value – Negative control optical density value)/(Positive control optical density value – Negative control optical density value). A cut-off S/P value of 0.4 has been determined by the manufacturers (ABIC Ltd, Israel) to indicate a sufficiently high antibody level to confer protective maternal immunity against coccidiosis and is used as a key parameter in the quality control of the commercial ELISA kit. As an example of our typical results, ten breeder flocks from various countries (six from Israel, two from South Africa, and one each from Argentina and Thailand) were monitored throughout their entire laying period (Figure 1). In these immunized flocks, the average ELISA S/P ratio was 0.8 or higher for the first 3 months post-immunization, dropped to 0.6 at 4 months and remained well above the ELISA kit cut-off S/P ratio of 0.4 throughout the hens' laying period. In contrast, control flocks had S/P values of less than 0.4 (0.18±0.1, average±S.D.) throughout the laying period of the hens' life, most often in the 0.1–0.2 range. We believe that the maintenance of the high specific IgG antibody levels in the vaccinated flocks is due to the boosting effect of oocysts that are naturally encountered in every chicken house worldwide. It is conceivable that, on some farms, natural exposure by itself can lead to increased maternal antibody levels, however, without vaccination, these natural titres are likely to be variable, unreliable and relatively non-specific.

In summary, these field trials on breeding hens demonstrate the long-term safety and immunogenicity of vaccination with purified gametocyte antigens from *E. maxima*. The antibody levels maintained in vaccinated flocks are sufficient to ensure the transmission of protective antibodies to offspring throughout the egg laying lifetime of the hens.

### Resistance to coccidiosis and the development of natural immunity by the progeny of hens vaccinated with purified gametocyte antigens

Commercial broiler breeder hens were vaccinated twice (at 15 and 20 weeks of age) with purified gametocyte antigens and eggs were collected from 43 week old hens and incubated to hatching. At different time points, the hatchlings were moved into clean cages and challenged with various doses of *E. tenella* oocysts, depending on the age of the hatchlings. Peak and total oocyst excretion, assessed by daily faecal oocyst counts, was used to determine resistance to infection induced by vaccination (see Table 2).

**Table 2:** The effect of maternal immunization with purified gametocyte antigens of *Eimeria maxima* on the development of resistance to *Eimeria tenella* in offspring chickens.

Age of challenge	Peak numbers of oocysts per gram faeces for vaccine birds (average±S.D.)×10^−3^ (n = 4 groups of 15 birds)	Peak numbers of oocysts per gram faeces for control birds (average±S.D.)×10^−3^ (n = 4 groups of 15 birds)	Peak % reduction in oocyst excretion by vaccine birds	Overall numbers of oocysts per gram faeces, days 4–14 post-challenge, for vaccinated birds (average±S.D.)×10^−3^ (n = 4 groups of 15 birds)	Overall numbers of oocysts per gram faeces, days 4–14 post-challenge, for control birds (average±S.D.)×10^−3^ (n = 4 groups of 15 birds)	Overall % reduction in oocyst excretion by vaccine birds
4 days	56.2±2.2	175.0±81.7	67.9	115.0±5.5	310.9±53.0	63.0
39 days	48.0±36.9	405.7±214.8	88.2	104.4±92.9	713.6±322.8	85.4
57 days	0.1±0.1	3.6±1.8	98.0	0.1±0.1	6.2±3.6	98.4

Commercial broiler breeder hens (14,532 vaccinated with purified gametocyte antigens and 7,256 control) were vaccinated twice with purified gametocyte antigens, (at 15 and 20 weeks of age). Eggs were collected from groups of vaccinated and unvaccinated 43-week old hens and incubated to hatching. At 4 days of age, four groups of 15 male chicks from vaccinated and unvaccinated hens were challenged with 50 oocysts of *E. tenella*. At 39 and 57 days of age four groups of 15 chicks from each of vaccinated and unvaccinated hens were challenged with 25,000 oocysts of *E. tenella*. Faeces were collected from days 4–14 after challenge infection and oocyst counts performed. Oocyst excretion is expressed in two ways: [i] by peak oocyst excretion, which is a measure of the highest number of oocysts per gram of faeces on a single day after challenge infection (generally day 7 post-challenge) for groups of 15 chickens; and [ii] overall oocyst excretion, which is a measure of the number of oocysts per gram faeces found in the total faecal collection for days 4 to 14 post-challenge for groups of 15 chickens. The differences for both the peak and total oocyst counts in the vaccinated and control groups at the three time points are all statistically significant at the p<0.05 level (one-way ANOVA, Student's t-test).

Unchallenged, 4 day old control chicks shed no detectable oocysts, as expected. Peak oocyst excretion by chicks from gametocyte antigen vaccinated hens was reduced by 67.9% compared with oocyst excretion by chicks from unvaccinated hens (significant at p<0.05, Student's t-test; Table 2), which is similar to our previous results in laboratory-based trials [5], [6], [7], [11]. A similar reduction was found in the comparison of total oocyst counts, where we observed a 63% reduction in the progeny of vaccinated hens (significant at p<0.05, Student's t-test; Table 2).

At 39 days of age, peak oocyst excretion by birds from purified gametocyte antigen vaccinated hens was reduced by 88.2% compared with oocyst excretion by chicks from unvaccinated hens (significant at p<0.05, Student's t-test; Table 2). The same type of experiment was conducted when the birds were 57 days old and, in this experiment, peak oocyst excretion by chickens from the vaccinated group was reduced by 98.0% (significant at p<0.05, Student's t-test; Table 2). Similar results were found in comparing total oocyst counts, where we found a 85.4% and 98.4% reduction at 39 days and 57 days of age, respectively (significant at p<0.05, Student's t-test; Table 2).

To sum up, we have shown that, not only does vaccination with purified gametocyte antigens confer protection against *Eimeria* species in the early life of chicks via maternal transfer of antibodies, it also facilitates the development of active immunity in older birds at 8 weeks of age. This is a significant milestone because meat birds (broilers) typically live for only 5–7 weeks before being slaughtered for market.

### Growth and performance of the progeny of hens vaccinated with purified gametocyte antigens

**Figure 2:**
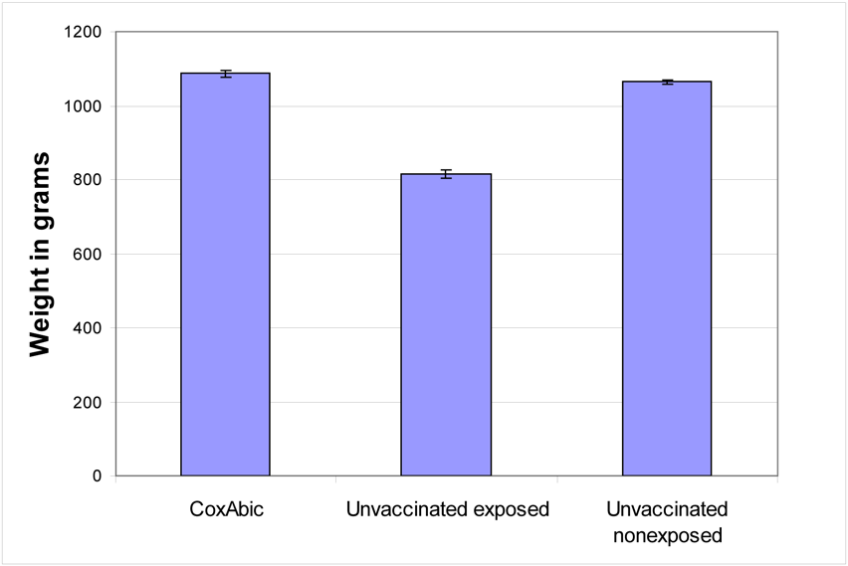
Effect of maternal immunization with purified gametocyte antigens of *Eimeria maxima* on weight loss due to infection with multiple species of *Eimeria* in progeny chickens. Commercial broiler breeder hens (14,532 vaccinated with purified gametocyte antigens and 7,256 control) were vaccinated twice with purified gametocyte antigens, (at 15 and 20 weeks of age). Eggs were collected from groups of vaccinated and unvaccinated 43 week old hens and incubated to hatching. Three different groups of 100 broiler chickens were raised: (A) chicks from unvaccinated hens, the chicks being raised in wire cages, with care being taken that they were not exposed to coccidian oocysts (i.e. non-exposed control group); (B) chicks from unvaccinated hens, the chicks being raised under normal commercial conditions on floor litter that was seeded with *Eimeria* oocysts; and (C) chicks from hens vaccinated twice with purified gametocyte antigens (CoxAbic), the chicks being raised under normal commercial conditions on floor litter that was seeded with *Eimeria* oocysts. When the birds were 10 days old, eight out of one hundred birds were orally infected with a cocktail containing 50 oocysts from each of four species of *Eimeria – E. maxima*, *E. tenella*, *E. acervulina* and *E. mitis*. Weekly checking of the litter for oocysts confirmed that the infections were successful in all groups of birds, with peaks of between 300,000 and 459,000 oocysts being found in every gram of floor litter. At 34 days of age, the birds were weighed. Results are means±S.E. Group B chicken weight was significantly lower than both Groups A and C (p<0.0001, one-way ANOVA, Student's t-test), which were not significantly different from each other.

Weight gain is the most important performance parameter for a chicken meat farmer and coccidiosis is recognised as one of the major causes of weight loss in broilers. To test the effect of maternal immunization with purified gametocyte antigens on growth of broiler chickens, we raised groups of 100 broilers of the Ross breed on litter that was deliberately seeded with oocysts of *E. maxima*, *E. tenella*, *E. acervulina* and *E. mitis* (see Figure 2). We chose this approach as a prelude to larger scale field trials because we can control the level of seeding of the litter to guarantee an exposure level that will have an affect on weight gain, whilst otherwise mimicking commercial conditions. We were also able to include negative control groups of chickens in these experiments, which was not possible for the large-scale field trials (see below).

At 34 days of age (ie, 24 days after exposure), the progeny of unvaccinated hens raised on litter weighed more than 200 grams less than either the progeny of vaccinated hens or the non-exposed birds, that is, an effective weight loss of more than 20% as a result of exposure to the four species of *Eimeria* (significant at p<0.0001, one-way ANOVA; Figure 2). The hatchlings from vaccinated hens were slightly, though not statistically significantly, heavier than the non-exposed birds, confirming that maternal immunization with purified gametocyte antigens protects broilers against one of the most serious effects of coccidiosis, namely, weight loss.

At 34 days of age, chickens were challenged by infecting individual birds with high doses of oocysts of one of the following species of *Eimeria*: *E. maxima*, *E. tenella*, *E. acervulina* or *E. mitis* (see Table 3). Oocyst counts were performed on faeces collected from individual birds on days 4–14 post infection and the birds were weighed 2 weeks after infection (i.e. at 49 days of age). As expected, birds from vaccinated and unvaccinated hens raised on floor litter (and, therefore, exposed to coccidial infection which induces active immunity) excreted greatly reduced numbers of oocysts of all species compared with the birds raised coccidia-free in wire cages – in both of these exposed groups of birds, whether challenged with *E. maxima*, *E. tenella*, *E. acervulina* or *E. mitis*, oocyst excretion was at least 97% less than that seen in naïve birds (data not shown).

**Table 3:** The effect of maternal immunization with purified gametocyte antigens of *Eimeria maxima* and natural immunity induced by exposure to oocysts on weight loss caused by various species of *Eimeria* in offspring chickens.

Challenge Species	A. Broilers from unvaccinated hens raised coccidia free (ie, in wire cages)		B. Broilers from unvaccinated hens raised with exposure to coccidia (ie, in floor litter)		C. Broilers from purified gametocyte antigen vaccinated hens, raised with exposure to coccidia (ie, on floor litter)	
	Average Weight±S.D. (g)	% Weight loss vs. unchallenged, unexposed broilers	Average Weight (g)	% Weight loss vs. unchallenged, unexposed broilers	Average Weight±S.D. (g)	% Weight loss vs. unchallenged, unexposed broilers
Unchallenged	2346±157	-	1791±367	23.6	2355±279	-
*E. tenella*	2176±210	7.2*	1869±214	20.3	2343±172	0.5
*E. acervulina*	2046±206	12.8*	1861±174	20.7	2231±245	5.3
*E. maxima*	1910±250	18.6*	1833±316	21.9	2151±175	8.6
*E. mitis*	1992±188	15.1*	1795±312	23.5	2189±313	7.0

Commercial broiler breeder hens were vaccinated and their offspring raised as described in the legend to Figure 2. At 34 days of age, the birds were divided into fourteen cages each containing five birds. The chickens were challenged by infecting individual birds with oocysts of one of the following species of *Eimeria*: *E. maxima* (25,000 oocysts), *E. tenella* (25,000 oocysts), *E. acervulina* (80,000 oocysts) or *E. mitis* (120,000 oocysts) with three replicates of five chickens per species. Two replicates of five chickens were left unchallenged (sham dosed with sterile water). The birds were weighed 2 weeks after infection. In Group A, challenge infection with every species of *Eimeria* caused a significant loss of weight versus unchallenged chickens (^*^ p<0.05; one-way ANOVA, Student's t-test). There was no significant weight loss in challenged birds in Group B or Group C versus unchallenged birds.

Infection with the various coccidia caused significant weight loss in the cage-reared, previously unexposed birds, varying from 7% in the case of *E. tenella* to 13% for *E. acervulina*, to 19% for *E. maxima* and 15% for *E. mitis* as compared to the unchallenged control group (significant at p<0.05, one-way ANOVA; Table 3). In contrast, the broilers from vaccinated hens that were floor-reared and previously exposed to *Eimeria* species, lost only between 0.5–8.6% of their weight (statistically these differences were not significant from the unchallenged group of progeny of vaccinated hens) as a result of challenge infection (Table 3). Broilers from unvaccinated hens that were previously exposed to *Eimeria* in the floor litter, did not show a significant loss of weight due to challenge, however, these broilers went through an infection during the seeding phase, immunizing them against reinfection. In addition, this unvaccinated, exposed group started out more than 200 grams lighter than their gametocyte antigen vaccinated counterparts and ended between 500 and 600 grams lighter (i.e. there was no weight compensation after the initial effect of the seeding with *Eimeria* oocysts in the control group). Thus, as a result of combined passive and active immunization, at 49 days of age the progeny of purified gametocyte antigen vaccinated hens were substantially heavier than both the cage-reared and floor-reared broilers from unvaccinated hens.

Our results showing protection against weight gain in small-scale floor-pen trials, led to large-scale field trials that were conducted on commercial farms in Brazil, Thailand, Israel and South Africa. As it is not possible to ask poultry farmers to compromise their profit margins by raising large numbers of broilers without any protection against coccidiosis, our large-scale field tests of the gametocyte antigen vaccine were evaluated against a “gold standard” positive control, either anti-coccidial drug incorporated into the feed of the broilers or a known, effective live vaccine regimen. Thus, our null hypothesis was that the growth and survival rates of offspring of hens vaccinated with purified gametocyte antigens are no less than the “gold standard” positive control chickens

The trials involved at least 1 million progeny of vaccinated hens and at least 1 million positive control birds in each country, allowing us to do a robust assessment of performance of these broiler chickens on four continents, with distinct climates and also different rearing conditions (eg, in Israel and South Africa, litter is always cleaned out between each flock, whereas in Brazil litter is occasionally reused). The trials that were carried out in Israel were particularly extensive, involving 60 million progeny of vaccinated hens and 112 million positive control birds.

Table 4 summarises the results in terms of country, age of slaughter, weight, feed conversion ratio (FCR = kg feed consumed/net weight gain; the lower the FCR the better), mortality and the EU performance index. The EU index was calculated using the following formula: (Body weight (g)×10)/(FCR×slaughter age in days) and gives a powerful measure of overall flock performance and profitability (the higher the EU index the better). In all trials, in all countries, the performance of offspring broilers from gametocyte antigen vaccinated hens was not significantly different to that of medicated or live vaccine control groups; this is true for all aspects of broiler growth. Thus, we must accept the null hypothesis that the growth and survival rates of progeny from hens vaccinated are the same as those of positive control chickens. Indeed, though the differences are not statistically different, in all but two trials (Fram B, Brazil and Farm A, Thailand) the EU Index was slightly higher in the progeny of vaccinated hens.

**Table 4:** The effect of maternal immunization with purified gametocyte antigens of *Eimeria maxima* on the growth performance of progeny broiler chickens raised under commercial conditions on farms in Brazil, Thailand, Israel and South Africa.

Country	Group	Number of Chickens	Age at Slaughter	Mortality (%)	FCR	Final Weight (g)	EU Index
**Brazil**							
Farm A							
	PGA	17,000	49	5.88%	2.090	2,810	259
	Drug	17,000	49	5.84%	2.180	2,680	237
Farm B							
	PGA	23,200	48	3.84%	1.770	2,720	311
	Drug	23,200	48	3.00%	1.810	2,790	315
Farm C*							
	PGA	1,300,000	46	4.48%	2.020	2,296	235
	Live vaccine	1,250,000	46	5.10%	2.020	2,296	233
**Thailand**							
Farm A*							
	PGA	1,205,760	44	2.40%	1.830	2.345	291
	Drug	3,259,017	44	2.75%	1.790	2.375	301
Farm B							
	PGA	346,800	45	2.30%	2.485	1.83	300
	Drug	225,600	45	2.50%	2.485	1.83	295
**Israel** **							
	PGA	60,000,000	45	5.48%	2.000	2.110	237
	Drug	112,000,000	45	5.73%	2.005	2.148	234
**South Africa**							
	PGA	755,000	38	4.33%	1.836	1.790	257
	Drug	795,000	38	4.42%	1.843	1.784	255
	PGA	137,280	38	3.60%	1.720	2.000	292
	Live vaccine	126,720	38	4.41%	1.760	1.970	281
	PGA	252,000	41	4.25%	1.88	1.880	234
	Live vaccine	276,000	41	5.00%	1.89	1.875	231

Commercial broiler breeder hens (Ross and Cobb breeds) were vaccinated twice with purified gametocyte antigens (PGA) at 15 and 20 weeks of age. Eggs were collected from groups of vaccinated and unvaccinated hens, incubated to hatching and broiler birds raised under normal commercial conditions for each country. Control flocks were either immunized with a commercial live vaccine or raised on feed containing anticoccidial chemoprophylactic drugs. There was no significant difference between the performance of control and vaccinated birds with respect to age at slaughter, mortality, Feed Conversion Rate (FCR), final body weight or European (EU) Performance Index (one-way ANOVA).

* These farms were from large integrations, and the results are from several chicken houses run over a period of 2–3 years.

** The results from Israel are the summary from several different farms run over a period of 5 years.

### Outcomes and General Conclusions

In the present study, involving 180 million broiler chickens, we have demonstrated the effectiveness of the first antiparasitic subunit vaccine of any kind used on a commercial scale worldwide. This vaccine contains antigens purified in native form from the gametocytes of *E. maxima*. It induces the production of high levels of protective IgG antibodies that are transferred from hens to egg yolks and, thence, to offspring broiler chickens. As a result of this maternal immunity, chickens can be raised without the need for any in-feed anti-coccidial drugs. This vaccination strategy reduces transmission between birds or, in other words, controls the level of overall infection in any given flock of chickens rather than preventing infection of individual birds, *per se*. It achieves this by stimulating production of antibodies that target proteins destined to be processed and cross-linked to form the oocyst wall of the parasite, a structure that is absolutely essential for the survival of the parasites outside of their host and, therefore, vital for transmission of parasites from host to host [2]. Importantly, because of the conserved, functional importance of oocyst wall formation in the parasites' lifecycle, immunization with the gametocyte antigens confers protection against a range of coccidial parasites even though it is composed of antigens from the gametocytes of *E. maxima* only. Another major benefit of this transmission-blocking immunity is that, in addition to the protection conferred by the maternal transfer of the IgG antibodies, individual chickens are additionally able to develop their own strong, natural immunity to the asexual stages of the parasite, which protects them in later weeks, well after maternal antibody levels have waned.

## Materials and Methods

### Production of purified gametocyte antigens

Purified gametocyte antigens were produced by ABIC Ltd, Bet Shemesh, Israel as described previously [6], and according to the manufacturer's standard operating procedures, from the highly enriched gametocyte stages of *Eimeria maxima*.

### Vaccination with purified gametocyte antigens

Affinity purified gametocyte antigens were emulsified in a water-in-oil emulsion and tested for sterility, stability and safety. Vaccine efficacy was tested by immunising Specific Pathogen Free chickens with a given batch and demonstrating by ELISA that the antibody titre induced was above the cut-off level required for providing protective immunity (see below). These tests were carried out at ABIC Ltd., Israel under the rules for animal ethics in accordance with Israeli law and those of the Veterinary Laboratory Agency, United Kingdom (the VLA is bound by the Animals (Scientific Procedures) Act 1986, which is administered by the Home Office, UK). Each 0.5 ml dose of vaccine contained 50 µg of gametocyte antigen. Hens were vaccinated twice by intramuscular injection - at 15 and 20 weeks of age – that is, just prior to the start of their laying period. All experiments were monitored by qualified scientists, veterinarians and the farmers themselves. Feed was provided by commercial companies that were instructed to clean out the chutes used for preparing our batch from any remaining anticoccidial drugs prior to preparation of the drug-free feed used in the trial. In order to be absolutely certain that no errors were made at the feed mill, the feed was tested for the presence (in the case of feed used as a drug containing control group) or absence (used for the vaccinated group) of the coccidiostats being used at that time in that particular feed mill. In the rare event there was an error made in preparing the feed, the experiment was aborted.

### Measurement of antibody levels

Sera were taken on the day of the first vaccination and then commencing 4 weeks post immunization. Sera were collected periodically until the end of the laying period and antibody levels measured by ELISA. The ELISA kit was produced by ABIC Ltd, Bet Shemesh, Israel, and was used exactly as recommended by the manufacturers. The kit contains ELISA plates coated with affinity purified macrogametocyte antigen from *E. maxima*, plus known positive and negative control sera and all the reagents needed for performing the test. The ELISA results are expressed as an S/P ratio, which is calculated as follows: (Sample optical density value – Negative control optical density value)/(Positive control optical density value – Negative control optical density value). A cut-off S/P value of 0.4 has been determined by the manufacturers to indicate a sufficiently high antibody level to confer protective maternal immunity against coccidiosis.

### Oocyst counts

Faecal oocyst counts or litter oocyst counts from samples collected weekly from individual chicken houses were performed as described previously [16]. For litter counts, samples were collected at a variety of points within the shed, the litter was well mixed, a sample was weighed, water was added and left overnight at 4°C. The sample was then mixed well, filtered through a strainer, spun at 1500 rpm for 5 minutes and brought back to the original volume with a saturated salt solution. Oocyst counting was performed using a McMaster flotation chamber. The number of oocysts per gram litter or faeces was calculated by dividing the number of oocysts counted microscopically by 0.15, multiplying by the dilution factor and then dividing by the weight in grams of the sample.

### Assessment of performance of chickens

For breeder farms, hens were kept in separate but very similar houses, and monitored for mortality, morbidity, egg laying and hatchability of chicks. Calculations were made as far as the number of eggs laid per hen and the number of chicks hatched per hen in the vaccinated versus control groups.

For the broiler chicks, houses were monitored for chicken mortality, weight gain and feed conversion. The feed conversion ratio (FCR) was calculated by Feed(kg)/BW(kg). The EU performance index was calculated using the following formula: (Body weight (kg)×10,000)/(FCR×slaughter age in days). All of the studies using commercial breeder and broiler chickens were carried out in accordance with commercial growing and animal experimentation rules governing their care & welfare in each of the countries where tests were performed (Thailand, India, South Africa, Israel, Argentina and Brazil).
